# Long COVID in children and adolescents: COVID-19 follow-up results in third-level pediatric hospital

**DOI:** 10.3389/fped.2023.1016394

**Published:** 2023-01-30

**Authors:** Lourdes María del Carmen Jamaica Balderas, Amairani Navarro Fernández, Susana Azeneth Dragustinovis Garza, María Isabel Orellana Jerves, Walter Ernesto Solís Figueroa, Solange Gabriela Koretzky, Horacio Márquez González, Miguel Klünder Klünder, Juan Garduño Espinosa, Jaime Nieto Zermeño, Mónica Villa Guillén, Rómulo Erick Rosales Uribe, Victor Olivar López

**Affiliations:** ^1^ Department of Pulmonology, Hospital Infantil de Mexico Federico Gomez Instituto Nacional de Salud, Mexico, Mexico; ^2^ Department of Clinical Research, Hospital Infantil de Mexico Federico Gomez Instituto Nacional de Salud, Mexico, Mexico; ^3^ General Direction, Hospital Infantil de Mexico Federico Gomez Instituto Nacional de Salud, Mexico, Mexico; ^4^ Department of Emergency Medicine, Hospital Infantil de Mexico Federico Gomez Instituto Nacional de Salud, Mexico, Mexico

**Keywords:** long covid, dyspnea, fatigue, Mexico, pediatrics

## Abstract

**Introduction:**

In children, the manifestations of coronavirus disease 2019 (COVID-19) in the acute phase are considered mild compared with those in adults; however, some children experience a severe disease that requires hospitalization. This study was designed to present the operation and follow-up results of the Post-COVID-19 Detection and Monitoring Sequels Clinic of Hospital Infantil de Mexico Federico Gómez in managing children with a history of SARS-CoV-2 infection.

**Methods:**

This was a prospective study conducted from July 2020 to December 2021, which included 215 children aged 0–18 years who tested positive for SARS-CoV-2 on polymerase chain reaction and/or immunoglobulin G test. The follow-up was conducted in the pulmonology medical consultation; ambulatory and hospitalized patients were assessed at 2, 4, 6, and 12 months.

**Results:**

The median age of the patients was 9.02 years, and neurological, endocrinological, pulmonary, oncological, and cardiological comorbidities were the most commonly observed among the patients. Moreover, 32.6% of the children had persistent symptoms at 2 months, 9.3% at 4 months, and 2.3% at 6 months, including dyspnea, dry cough, fatigue, and runny nose; the main acute complications were severe pneumonia, coagulopathy, nosocomial infections, acute renal injury, cardiac dysfunction, and pulmonary fibrosis. The more representative sequelae were alopecia, radiculopathy, perniosis, psoriasis, anxiety, and depression.

**Conclusions:**

This study showed that children experience persistent symptoms, such as dyspnea, dry cough, fatigue, and runny nose, although to a lesser extent than adults, with significant clinical improvement 6 months after the acute infection. These results indicate the importance of monitoring children with COVID-19 through face-to-face consultations or telemedicine, with the objective of offering multidisciplinary and individualized care to preserve the health and quality of life of these children.

## Introduction

Infection with SARS-CoV-2 has caused significant mortality and morbidity worldwide (1). Pediatric cases are estimated to account for 11.8% (0–17 years), 15.6% (0–18 years), and 10% (0–19 years) of all cases in the European Union, particularly Italy and Denmark. Coronavirus disease 2019 (COVID-19)-related deaths in children remain rare, with a rate of 0.17 of 100,000 habitants (2). In Mexico, until January 2022, 230,900 children aged <18 years have been registered to have confirmed COVID-19 with 1,013 deaths, mostly affecting children aged <1 year (27%), followed by those aged 15–17 years (23%) (3). Those numbers demonstrate the importance of identifying the clinical course of the disease in children. After 2 years since the start of the pandemic and after several publications on the epidemiology, pathogenicity, and clinical manifestations of the disease, uncertainty remains about the long-term effects that this may produce in the pediatric population (4). In children, the manifestations of COVID-19 in the acute phase are considered mild compared with those in adults (5, 6); however, some children experience a severe disease that requires hospitalization, with a worse prognosis because of comorbidities (7, 8). According to the National Institute for Health and Care Excellence guidelines, long COVID is defined as the persistence of symptoms for more than 12 weeks after SARS-CoV-2 infection, characterized by respiratory, cardiac, rheumatological, dermatological, neurological, and ophthalmological manifestations, such as multisystemic inflammatory syndrome (MIS-C), which require early detection and timely intervention (9, 10). Unlike what happens in adults, considering that pediatric patients are in the stage of growth and development is important; therefore, the consequences of the acute disease, as well as its sequelae or persistent symptoms observed weeks later, could negatively affect this stage. According to studies involving the pediatric population, it has been described that between 4% and 66% of children who test positive for SARS-CoV-2 experience symptoms of long COVID (1), in contrast to case–control studies that estimated a prevalence rate ranging between 2% and 3.5% (11). Despite the frequency and duration differences between studies, the most common symptoms reported in children and adolescents were headache (3%–80%), fatigue (3%–87%), sleep disturbances (2%–63%), difficulty concentrating (2%–81%), abdominal pain (1%–76%), and myalgia or arthralgia (1%–61%) (12).

Hospital Infantil de México Federico Gómez is considered a third-level center that promotes the advancement of pediatrics and development of research and provides highly specialized medical care for the prevention, diagnosis, and treatment of diseases. During this pandemic, Hospital Infantil de México Federico Gómez became a COVID-19 reference center for pediatric patients who required care and hospitalization for moderate and severe COVID-19. On July 14, 2020, the Pulmonology Department founded the Post-COVID-19 Detection and Monitoring Sequels Clinic with the objective of the timely detection of pulmonary and systemic sequelae in the median and short terms and the assurance of the continuity of multidisciplinary management and follow-up with different specialties that the institute offers to improve the quality of life and facilitate the progressive return to normality. This study was designed to present the operation and follow-up results of the Post-COVID-19 Detection and Monitoring Sequels Clinic of Hospital Infantil de Mexico Federico Gómez in terms of managing children with a history of SARS-CoV-2 infection.

## Materials and methods

The Post-COVID-19 Detection and Monitoring Sequels Clinic of Hospital Infantil de Mexico Federico Gómez conducted a prospective study from July 2020 to December 2021. The information was collected during direct interrogation in the consultations. The study included 215 children aged 0–18 years who tested positive for SARS-CoV-2 on polymerase chain reaction (PCR) and/or immunoglobulin G test. Those without a confirmatory SARS-CoV-2 infection test or those with an incomplete questionnaire were excluded from this study. The Ethics Committee of Hospital Infantil de Mexico Federico Gómez reviewed and approved the study protocol (approval No. HIM-2020-048).

For this study, the following definitions were established: long COVID: the persistence of symptoms for more than 12 weeks after acute SARS-CoV-2 infection (12); mild cases: upper airway symptoms without pneumonia; moderate cases: mild pneumonia without acute respiratory insufficiency; severe cases: pneumonia with respiratory insufficiency that requires noninvasive ventilation; and critical cases: respiratory failure requiring mechanical ventilation or the presence of shock or organ failure (13).

The protocol for post-COVID-19 evaluation and follow-up was realized in the pulmonology medical consultation, where ambulatory and hospitalized patients were assessed at 2, 4, 6, and 12 months.

At the first consultation (2 months), the following procedures were performed:

- The severity of SARS-CoV-2 acute infection was identified.
- A questionnaire was applied, which included demographic characteristics, comorbidities, contagion contact, symptoms during diagnosis, imaging evaluation (chest x-ray and high-resolution chest computed tomography), laboratory tests, treatment, and complications.
- The current clinical status of the patients was evaluated by interrogation, physical exploration, and complementary studies.
- Laboratory control tests were requested, including hematic biometry, blood chemistry, coagulation times, fibrinogen, ferritin, and C-reactive protein.
- Pulmonary function tests were performed (e.g., spirometry, plethysmography, and diffusing capacity of the lungs for carbon monoxide).
- Pulmonary rehabilitation, nutrition, psychology, phoniatrics, and audiology assessments were performed.
- Opportune reference to other subspecialties according to symptoms was initiated.

For the subsequent consultations (4, 6, and 12 months), the following procedures were performed:

- A questionnaire was applied, and data were recollected.
- The current clinical status of the patients was evaluated, searching for persistent symptoms.
- Pulmonary rehabilitation assessment was performed, including the 6-minute-walk and Ruffier tests.
- Nutrition, psychology, phoniatrics, and audiology were followed up.
- In cases with sequelae, opportune management, assessment, closer appointments were performed at 8, 10, and 12 months.

All statistical analyses were performed using Statistical Package for the Social Sciences, version 20.0. Categorical variables were summarized using absolute frequencies and percentages. Meanwhile, continuous variables were reported as means ± standard deviations or medians and interquartile ranges (IQRs) according to data distribution. Normality was evaluated using the Kolmogorov–Smirnov test. Differences between categorical variables were evaluated using the chi-square test or Fisher's exact test, as appropriate.

## Results

From July 2020 to December 2021, 215 pediatric patients were included, of whom 53% were males and 47% were females, with a median age of 9.02 years (IQR, 18). Of the 215 patients, 10.7% were infants, 7% were toddlers, 17.7% were preschoolers, 27.4% were school-age children, and 37.2% were adolescents.

Moreover, 67.4% of the patients had neurological (8.8%), endocrinological (7.9%), pulmonary and oncological (7.4%), and cardiological (5.6%) comorbidities, and 14.9% had genetic, dermatological, urological, and orthopedic comorbidities. Of the 215 patients, 50.7% had mild disease, 26% had moderate disease, 14.9% had severe disease, and 8.4% had MIS-C (Multisystem Inflammatory Syndrome in Children) (Table 1).

**Table 1:** Etiological groups associated with COVID-19 severity.

	Classification				Total
	Mild COVID	Moderate COVID	Severe COVID	MIS-C^a^	
	Count (%)	Count (%)	Count (%)	Count (%)	(%)
Previously healthy	27 (12.5)	17 (7.9)	15 (7)	11 (51)	70 (32.6)
Cardiology	10 (4.7)	0	2 (0.9)	0	12 (5.6)
MIPneumology	8 (3.7)	4 (1.9)	3 (1.4)	1 (0.5)	16 (7.4)
Oncology	8 (3.7)	7 (3.2)	0	1 (0.5)	16 (7.4)
Hematology	1 (0.5)	1 (0.5)	1 (0.5)	0	3 (1.4)
Nephrology	3 (1.4)	4 (1.9)	1 (0.5)	0	8 (3.7)
Rheumatology	2 (0.9)	0	1 (0.5)	1 (0.5)	4 (1.9)
Gastroenterology	7 (3.2)	3 (1.4)	0	0	10 (4.6)
Allergies	1 (0.5)	2 (0.9)	0	2 (0.9)	5 (2.3)
Endocrinology	2 (0.9)	7 (3.2)	7 (3.2)	1 (0.5)	17 (7.9)
Surgical	2 (0.9)	0	1 (0.5)	0	3 (1.4)
Neurology	10 (4.6)	8 (3.7)	1 (0.5)	0	19 (8.8)
Others	28 (13)	3 (1.4)	0	1 (0.5)	32 (14.8)
Total	109 (50.6)	56 (26)	32 (14.8)	18 (8.3)	215 (100)

^a^ MIS-C: Multisystem Inflammatory Syndrome in Children.

The main symptoms during diagnosis were fever (61.9%), headache (36.7%), rhinorrhea (27.9%), abdominal pain (27.4%), dyspnea (24.2%), and dry cough (21.4%). The first consultation (2 months) showed an improvement of the persistent symptoms as follows: dyspnea at 8.8%, dry cough at 6.5%, fatigue at 6.5%, and runny nose at 5.1%. In the second consultation (4 months), symptoms, such as runny nose (2.8%), dyspnea, fatigue, and dry cough (2.3%), persisted. In the third consultation (6 months), four patients continued to have dyspnea (1.9%), whereas, in the fourth consultation (8 months), only one patient continued to have headache and runny nose (0.5%) (Table 2).

**Table 2:** Symptoms at diagnosis and follow-up consultations.

	Consultation					
	COVID diagnosis	First consultation	Second consultation	Third consultation	Fourth consultation	
	Count (%)	Count (%)	Count (%)	Count (%)	Count (%)	*p* ^a^
Headache	79 (36.7)	5 (2.3)	0	0	1 (0.5)	<0.001
Fever	133 (61.9)	3 (1.4)	0	0	0	<0.001
Rhinorrhea	60 (27.9)	11 (5.1)	6 (2.8)	1 (0.5)	1 (0.5)	<0.001
Dyspnea	52 (24.2)	19 (8.8)	5 (2.3)	4 (1.9)	0	<0.001
Dry cough	46 (21.4)	14 (6.5)	5 (2.3)	1 (0.5)	0	<0.001
Wet cough	13 (6)	4 (1.9)	1 (0.5)	0	0	<0.001
Odynophagia	25 (11.6)	1 (0.5)	1 (0.5)	0	0	<0.001
Desaturation	37 (17.2)	3 (1.4)	2 (0.9)	0	0	<0.001
Conjunctival injection	33 (15.3)	1 (0.5)	0	0	0	<0.001
Chemosis	3 (1.4)	0	0	0	0	0.358
Epiphora	6 (2.8)	1 (0.5)	0	0	0	0.197
Irritability	34 (15.8)	1 (0.5)	0	0	0	<0.001
Abdominal pain	59 (27.4)	3 (1.4)	1 (0.5)	0	0	<0.001
Diarrhea	45 (20.9)	3 (1.4)	0	0	0	<0.001
Nausea and/or vomiting	42 (19.5)	2 (0.9)	1 (0.5)	0	0	<0.001
Smell alteration	29 (13.5)	5 (2.3)	0	0	0	<0.001
Taste alteration	23 (10.7)	3 (1.4)	0	0	0	<0.001
Skin alterations	8 (3.7)	2 (0.9)	1 (0.5)	0	0	0.306
Vasculitis	2 (0.9)	0	0	0	0	0.573
Fatigue	42 (19.5)	14 (6.5)	5 (2.3)	0	0	<0.001
Myalgia	42 (19.5)	2 (0.9)	0	1 (0.5)	0	<0.001
Paresthesia	6 (2.8)	2 (0.9)	0	0	0	0.364
Rash	29 (13.5)	1 (0.5)	0	0	0	<0.001
Joint pain	23 (10.7)	6 (2.8)	0	0	0	<0.001

^a^ Chi-square.

Then, 63.7% required In-patient treatment, 25.5% requires an intensive care unit support, and 14.4% were discharged but were oxygen-dependent.

Regarding diagnostic imaging studies, 27.9% of the patients presented with an interstitial pattern, 21.4% had an alveolar pattern, and 23.7% presented with bilateral involvement. In the second evaluation, 13.5% continued to present with an interstitial pattern and 0.9% continued to present with alveolar pattern, with the same predominance of bilateral involvement (Table 3).

**Table 3:** Radiological alteration at diagnosis and follow-up consultations.

	Consultation				
	COVID diagnosis	Second consultation	Third consultation	Fourth consultation	
	Count (%)	Count (%)	Count (%)	Count (%)	*p* ^a^
Interstitial pattern	60 (27.9)	29 (13.5)	3 (1.4)	0	0.816
Alveolar pattern	46 (21.4)	2 (0.9)	1 (0.5)	0	<0.001
Air trapping	8 (3.7)	5 (2.3)	0	1 (0.5)	0.001
Unilateral involvement	5 (2.3)	1 (0.5)	1 (0.5)	0	0.408
Bilateral involvement	51 (23.7)	16 (7.44)	1 (0.5)	0	0.256
Peripheral distribution	7 (3.2)	0	0	0	0.275
Pleural effusion	7 (3.2)	0	0	0	0.268

^a^ Chi-square.

Regarding tomographic findings during the acute phase, 6.9% of the patients presented with ground-glass opacities with overlapping consolidation, 4.1% had pleural effusion and ground-glass opacities, 2.8% showed air bronchogram, and only 0.5% presented with pulmonary fibrosis. Meanwhile, in the second evaluation, 1.5% continued to show ground-glass opacities (Table 4).

**Table 4:** Tomographic alteration at diagnosis and follow-up consultation.

	Consultation				
	COVID diagnosis	First consultation	Second consultation	Third consultation	
	Count (%)	Count (%)	Count (%)	Count (%)	p^a^
Pleural effusion	9 (4.1)	0	0	1 (0.5)	0.005
Halo sign	1 (0.5)	0	1 (0.5)	0	0.273
Pulmonary fibrosis	1 (0.5)	0	0	0	0.278
Isolated pleural thickening	4 (1.9)	0	1 (0.5)	1 (0.5)	0.090
Pericardial effusion	1 (0.5)	0	0	0	0.278
Ground-glass opacities	9 (4.1)	1 (0.5)	3	0	0.095
Consolidation without ground-glass	5 (2.3)	0	1 (0.5)	1 (0.5)	0.069
Pulmonary vascular dilatation	1 (0.5)	0	0	0	0.278
Air bronchogram	6 (2.8)	0	0	0	0.030
Bronchial wall thickening	3 (1.4)	0	0	0	0.125
White lung status	1 (0.5)	0	0	0	0.273
Parenchymal fibrotic bands	2 (0.9)	0	1 (0.5)	0	0.298
Ground-glass opacities with overlapping consolidation	15 (6.9)	1 (0.5)	3	0	0.010
Predominant consolidated pattern	4 (1.9)	0	0	0	0.080
Linear opacities	1 (0.5)	0	0	0	0.332
Crazy pavement	3 (1.4)	0	0	0	0.125
Melted sugar sign	1 (0.5)	0	0	0	0.278

^a^ Chi-square.

Regarding spirometry findings, 2.7% of the patients presented with an obstructive pattern, 0.4% showed findings suggestive of restriction, 0.9% had evidence of air trapping on plethysmography, and 0.4% showed a decrease in diffusing capacity of the lungs (DLCO) and maximal inspiratory and expiratory pressures (PImax and PEmax).

Of the 215 patients, 6.5% presented with pulmonary arterial hypertension (PAH), 3.3% showed pericardial effusion, and 2.8% had left ventricular dysfunction. In the second evaluation, the proportion of patients presenting with PAH, pericardial effusion, and aneurysm decreased by 0.9%, with a further decrease of 0.5% in the third evaluation.

The main acute complications were severe pneumonia (14.8%), coagulopathy (3.7%), nosocomial infections (1.9%), acute renal injury (1.3%), cardiac dysfunction (1.3%), and pulmonary fibrosis (0.5%). The more representative sequelae were alopecia (2.3%), radiculopathy (1%), perniosis (0.5%), psoriasis (0.5%), anxiety (0.5%), and depression (0.5%) (Table 5).

**Table 5:** Complications and sequelae associated with COVID-19 severity.

	Classification			
	Mild COVID	Moderate COVID	Severe COVID	MIS-C^a^
	Count (%)	Count (%)	Count (%)	Count (%)
Nosocomial infection	1 (0.5)	0	3 (1.4)	1 (0.5)
Radiculopathy	1 (0.5)	0	1 (0.5)	0
Liver disease	1 (0.5)	0	0	0
Acute kidney injury	0	1 (0.5)	1 (0.5)	1 (0.5)
Coagulopathy	2 (0.9)	1 (0.5)	3 (1.4)	2 (0.9)
Heart failure	0	1 (0.5)	1 (0.5)	1 (0.5)
Pneumothorax	0	1 (0.5)	1 (0.5)	0
Pulmonary fibrosis	0	0	1 (0.5)	0
Exanthema erythematous	0	0	0	2 (0.9)
Perniosis	1 (0.5)	0	0	0
Hair loss	0	1 (0.5)	2 (0.9)	2 (0.9)
Ketoacidosis	0	1 (0.5)	0	0
Behavior change	0	0	1 (0.5)	0
Anxiety	0	1 (0.5)	0	0
Depression	0	1 (0.5)	0	0
Thrombosis	0	0	1 (0.5)	0
D-dimer elevation	0	0	0	2 (0.9)

^a^ MIS-C, Multisystem Inflammatory Syndrome in Children.

## Discussion

This study showed that on third of the children had persistent symptoms at 2 months, including dyspnea, dry cough, fatigue, and runny nose. These findings are consistent with those reported in other pediatric studies, where between 8% and 58% of children who test positive for SARS-CoV-2 experience symptoms of long COVID. However, because a control group was not included, there is a risk of overestimating the prevalence of long COVID (2). In a prospective study in the United Kingdom, where 1,735 cases and controls were included, a prevalence rate of 3.5% was reported (14). Moreover, a Danish study that included 37,522 children aged 0–17 years who tested positive for SARS-CoV-2 on PCR and 78,037 randomly selected controls who tested negative for SARS-CoV-2 reported a prevalence rate of 2.8% in children aged 0–5 years and 0.8% in children aged 6–17 years [2)]. These findings reflect the need to conduct a study with controls in our setting to predict more reliable prevalence.

In this study, the main persistent symptoms were dyspnea, runny nose, cough, and fatigue. In contrast, in a meta-analysis involving pediatric and adult individuals, the most common persistent signs and symptoms were weakness, malaise, fatigue, and impaired concentration (15). In a study involving 236 children with a history of COVID-19 infection, Leva Rog et al. reported persistent irritability in 24.3%, runny nose in 16.1%, cough in 14.4%, dyspnea on exertion in 7.2%, and dyspnea at rest in 4.7% (16). Ismail M Osmanov et al., through a prospective cohort of 518 pediatric patients, found that a quarter of the children and adolescents had persistent symptoms, including fatigue (10.6%), sleep disturbance (7.2%), taste and smell disturbances (6.2%), gastrointestinal problems (4.4%), dermatological issues (3.6%), neurological problems (3%), respiratory disturbances (2.5%), cardiovascular problems (1.9%) and musculoskeletal issues (1.8%), suggesting a discrepancy between the pediatric and adult population. This difference can be explained because one-third of our population was under six years old and, by 2021, different variants of SARS-CoV-2 were added that were identified in Mexico, but unfortunately we do not have the analysis of variants for this study, which could be identified as a limitation, but it is undoubtedly a topic for future research. According to international publications, respiratory symptoms tend to persist with recovery in a period of 1–5 months (17), as shown in our study, where at 6 months, only one patient continued to experience symptoms.

It was observed that previously healthy individuals, although to a lesser extent, can still present with severe disease and MIS-C, in contrast to patients with comorbidities where neurological (epilepsy), endocrinological (obesity), and pulmonary (asthma, allergic rhinitis, and bronchopulmonary dysplasia) pathologies predominate, which is explained by the high number of patients with uncontrolled seizures, obesity, and asthma (10.2%). A study conducted in Lithuania involving a cohort of pediatric patients revealed that among patients with comorbidities, 7.2% had asthma and 2.1% had epilepsy (16). Likewise, hospitalized patients more frequently present with persistent symptoms in the first evaluation, so that more than half of the patients with dyspnea and fatigue have a history of in-hospital management; however, persistent symptoms were reported in outpatients with mild disease, suggesting that long COVID is a syndrome that affects both previously hospitalized and non-hospitalized patients (15), as indicated by a prospective study of persistent symptoms 6 months after an acute infection, where a higher incidence of anosmia/dysgeusia was reported in patients with mild disease and fatigue, dyspnea, and neurological disorders in patients with severe disease (18). It is important to emphasize the parent's commitment to the protocol of attending face-to-face check-ups despite the restrictions due to the pandemic; however, it was observed that some parents of patients with mild COVID did not show up for consultation due to anxiety and fear of reinfection, which could reduce the cases of mild COVID in our study, contrary to the parents of patients with severe COVID, where post-traumatic stress and anxiety about the severity of the disease led them to a greater commitment to the study in question. As demonstrated by the study by A. Orsini, which refers to higher symptoms of post-traumatic stress, anxiety, and depression among parents whose children tested positive for COVID-19 with respect to parents whose children tested negative during the acute phase of the pandemic, exacerbate by quarantine and economic loss, which also have a significant negative impact on the mental health of the parents (19).

Regarding imaging studies, chest x-ray showed greater predominance of interstitial rather than alveolar infiltrates, without distinguishing the severity of the disease as a factor for remaining radiological alterations, suggesting that an alveolar pattern is mostly associated with the acute phase of the disease with subsequent improvement after the convalescent period, compared with chest tomography, where ground-glass opacities persisted more frequently in patients with severe SARS-CoV-2 infection. These finding coincide with a cross-sectional study that included 57 adult patients who were evaluated 3 months after acute SARS-CoV-2 infection, where ground-glass opacities were observed more frequently, being significantly higher in hospitalized patients than in outpatients (20).

In altered pulmonary function tests, the obstructive pattern and decreased DLCO prevailed. A systematic review and meta-analysis on the follow-up of persistent symptoms and pulmonary function in post-COVID patients observed decreased oxygen diffusion capacity and lung volume restriction more frequently and airflow obstruction less frequently (21); however, these data pertain to the adult population. Another retrospective study that included 29 children reported the presence of an obstructive pattern on spirometry with air trapping in plethysmography more frequently (6). Nevertheless, more prospective studies involving the pediatric population with a larger sample size are necessary for greater reliability.

The main acute complications were severe pneumonia, nosocomial infection, coagulopathy, acute kidney injury, and heart failure, which were shown to be associated with critical illness, in contrast to sequelae, which are defined as residual effects occurring after the acute phase, including alopecia, radiculopathy, perniosis, psoriasis, anxiety, and depression. These sequelae were not related to the severity of the disease, except for pulmonary fibrosis, which was shown to be associated with critical illness, in addition to a longer stay in the intensive care unit, longer hospitalization time, and increased inflammatory load mediated by analytical parameters (22). In the cohort study by Ieva Roge et al., behavioral changes, irritability, and difficulty concentrating were reported as the main sequelae (16); there is a discrepancy in the prevalence of neurological and dermatological sequelae when excluding cases of pulmonary fibrosis. In our experience, a previously healthy individual with severe disease who required management in the intensive care unit with ventilatory support for 54 days and the need for a tracheostomy underwent chest computed tomography, which showed the presence of a crazy-paving pattern and parenchymal fibrotic bands; clinical improvement was achieved over time, and the patient is currently without oxygen dependence or tracheostomy. The patient's latest computed tomography showed limited areas of pulmonary fibrosis and persistent ground-glass opacities. This generates uncertainty if, according to the variants, there will be greater sequelae or pulmonary affection, which should be studied in the future.

Children with a history of SARS-CoV-2 infection require close surveillance, as evidenced by the study by Karin Magnusson et al. where they examined the need for medical attention of 10,279 children with a history of positivity for SARS-CoV-2 on PCR compared with 275,859 with negative PCR. They found that patients aged 1–5 years who tested positive had 2–3 times greater probability of requiring primary care for respiratory conditions, and they reported an immediate increase in the use of medical care during the first month after a positive result, lasting from 3 to 6 months in children aged 1–5 years and from 1 to 3 months in children aged 6–19 years (23). According to the results, it is shown that medical care does not end at the time of hospital discharge; instead, a multidisciplinary approach is required for developing future plans to provide comprehensive care to all patients (23). Thus, the Pulmonology Service of the Hospital Infantil de México Federico Gómez founded the clinic for detecting and monitoring post-COVID-19 sequelae, consisting of pulmonary rehabilitation, cardiology, phoniatrics, audiology, nutrition, psychology, and psychiatry services, among others, responsible for the evaluation and follow-up of each patient to identify persistent symptoms, complications, and sequelae involving long COVID (24). Therefore, according to the aforementioned logistics, the first post-COVID-19 clinic for the pediatric population in Mexico is presented as an incentive for the continuous evaluation of patients with a history of COVID-19 infection through outpatient clinics in the second and third levels of care.

This study has several limitations. First, the number of studies on long COVID in children reported in the world literature is limited because long COVID is a recently known entity. The second limitation is the lack of standardized and validated tools to reduce the variability of COVID-19 research reports and loss of patients due to omission of reference and lack of continuity due to family, economic, and pandemic losses. In the future, it will be necessary to conduct a cohort study to reduce the risk of overestimating the prevalence of long COVID in children and to analyze the variants involved to determine whether the incidence and severity of long COVID are associated with any specific variant.

## Conclusion

Hospital Infantil de México Federico Gómez is a COVID reference center, with a high number of patients with COVID-19; the Pulmonology Service decided to form a clinic for detecting and monitoring post-COVID-19 sequelae, the first of its kind in Mexico, with the aim of clarifying the clinical evolution of these patients and identifying persistent symptoms, sequelae, and complications that affect this population and their impact on quality of life. Our study showed that children can present persistent symptoms, such as dyspnea, dry cough, fatigue, and runny nose, although to a lesser extent than adults, with significant clinical improvement 6 months after the acute infection. The main acute complications were found to be severe pneumonia, nosocomial infection, coagulopathy, acute kidney injury, and heart failure; moreover, the sequelae observed included hair loss, radiculopathy, perniosis, psoriasis, depression, and anxiety. After 2 years, the importance of monitoring children with COVID-19 through face-to-face consultations or telemedicine, which allow an adequate questioning and complete physical examination, is emphasized because the use of online surveys represents a risk of bias due to the lack of objective clinical evaluations. Regardless of age, comorbidity, and severity of the disease, it is necessary to offer multidisciplinary and individualized care to preserve the health and quality of life of children with COVID-19.

## Data Availability

The raw data supporting the conclusions of this article will be made available by the authors, without undue reservation.
